# Infrastructural Aspects of Rain-Related Cascading Disasters: A Systematic Literature Review

**DOI:** 10.3390/ijerph17145175

**Published:** 2020-07-17

**Authors:** Thomas J. Huggins, Feiyu E, Kangming Chen, Wenwu Gong, Lili Yang

**Affiliations:** 1 Department of Statistics & Data Science, Southern University of Science and Technology, Shenzhen 518055, China; tjhuggins@uic.edu.hk (T.J.H.); u3005658@hku.hk (F.E.); 11849391@mail.sustech.edu.cn (K.C.); 11849396@mail.sustech.edu.cn (W.G.); 2 Division of Science & Technology, BNU-HKBU United International College, Zhuhai 519085, China

**Keywords:** cascading disasters, rain, infrastructure, mechanisms, systematic literature review

## Abstract

Cascading disasters progress from one hazard event to a range of interconnected events and impacts, with often devastating consequences. Rain-related cascading disasters are a particularly frequent form of cascading disasters in many parts of the world, and they are likely to become even more frequent due to climate change and accelerating coastal development, among other issues. (1) *Background*: The current literature review extended previous reviews of documented progressions from one natural hazard event to another, by focusing on linkages between rain-related natural hazard triggers and infrastructural impacts. (2) *Methods*: A wide range of case studies were reviewed using a systematic literature review protocol. The review quality was enhanced by only including case studies that detailed mechanisms that have led to infrastructural impacts, and which had been published in high-quality academic journals. (3) *Results*: A sum of 71 articles, concerning 99 case studies of rain-related disasters, were fully reviewed. Twenty-five distinct mechanisms were identified, as the foundation for a matrix running between five different natural hazards and eight types of infrastructural impacts. (4) *Conclusion*: Relatively complex quantitative methods are needed to generate locality-specific, cascading disaster likelihoods and scenarios. Appropriate methods can leverage the current matrix to structure both Delphi-based approaches and network analysis using longitudinal data.

## 1. Introduction

The devastating impacts of disasters such as the Odisha Super Typhoon of 1999, Hurricane Katrina in 2005, and the Central European floods of 2013 have highlighted widespread vulnerabilities to extreme weather events. These types of events involve wind speed, rainfall, and other meteorological variables that “exceed a particular threshold and deviate significantly from mean climate conditions” [1] (p. 2). They can also trigger further and even more catastrophic events, such as landslides and storm surge [2].

Progressions from an initial trigger to a range of subsequent disasters are commonly referred to as *cascading disasters*, which can include much broader and more severe impacts than the initial trigger event [3]. The 2019 Global Assessment Report on Disaster Risk Reduction [4] stated that “Cascading hazard processes refer to a primary impact (trigger) such as heavy rainfall, seismic activity, or unexpectedly rapid snowmelt, followed by a chain of consequences that can cause secondary impacts” (p. 49). For example, Hurricane Katrina triggered a 7.3 to 8.5 me storm surge that was combined with ongoing rainfall to inundate 80 percent of New Orleans’ urban infrastructure footprint [5,6]. Without well-informed interventions, the kinds of cascading impacts experienced during Hurricane Katrina are only likely to worsen in the face of accelerating climate change [7], increasingly complex interdependencies, environmental degradation [8], and rapid urban development in areas prone to meteorological hazards [5,9]. There is therefore a pressing need to better understand the secondary hazard events triggered by extreme weather, to better mitigate and prepare for a wider scope of relevant impacts.

Many of these secondary hazard events involve major infrastructure, such as power, electricity, and water supplies. As outlined by Pescaroli and Alexander [3], “critical infrastructure and complex adaptive systems may be the drivers that amplify the impacts of the cascade” (p. 2250). This makes infrastructural vulnerabilities and resilience a very important aspect of analyzing and managing cascading risks, alongside other complexities [3]. Focusing on infrastructural aspects of cascading disasters also helps address the risk of *Natech* events, where natural hazards trigger severe technological hazards, such as chemical spills [6] and cascading system failures [4]. These types of events can cause major disruptions to affected populations and to emergency response agencies, even when they do not amount to a disaster. Definitively disastrous Natech events, like those associated with the 2008 Wenchuan and the 2011 Great East Japan earthquakes, have had even more severe impacts on human health and economies, in addition to environmental damage [4].

When relevant links between natural and infrastructural hazard events are specified, damage assessments and predictions can reflect a broader and more accurate set of disaster impacts. As highlighted by Hillier, Macdonald, Leckebusch, and Stavrinides [10], the sum of these impacts extends well beyond standard measures of direct property damage and fatalities. Their analysis of weather-related hazard linkages was based on 124 years of meteorological and insurance-related data from the United Kingdom. Hillier et al. [10] found that estimates for direct economic impacts increased by 26 percent, when including statistically weighted linkages between hazard types rather than calculating the impacts associated with a single trigger.

This approach to analysis also permits emergency management agencies to better address relevant linkages, to prevent or mitigate downstream hazard events well before they occur. This reflects the generally substantial cost-effectiveness of hazard mitigation outlined by Kelman [11], for complementing more reactive aspects of emergency management such as emergency response. For example, sandbags are stored close to elevators prone to subterranean flooding in Shenzhen, China. These sandbags are deployed in front of elevators during heavy rainfall, rather than waiting for the shafts to flood, and for many thousands of elevators throughout the city to fail.

The current paper contributes to cascading disaster risk assessment by determining: 1. Known infrastructural impacts triggered by rain-related natural hazards, and 2. The mechanisms explaining linkages between each identified impact and trigger. This was achieved by systematically reviewing case studies of rainfall-related triggers, infrastructural impacts and mechanisms, before adding the results to a preceding review of natural hazard linkages by Gill and Malamud [2]. The combined matrix resulting from the current review provides a robust set of parameters for further analyses of cascading rain-related disaster risk by highlighting a broader, but nonetheless defined range, of known scenario elements.

The remainder of this Section 1 outlines challenges for the numerical analysis of cascading disaster risk, before explaining how case study reviews can help address those challenges. This is followed by Section 2 detailing the systematic literature review process used by the current research, to review a wide range of rain-related disaster case studies. Section 3 outlines how literature review results were used to develop a conceptual matrix of documented linkages between natural hazards and infrastructural impacts during cascading disasters, together with a list of associated mechanisms. Section 4 then compares these results and their limitations with prior research. This is followed by Section 5 that summarizes all the preceding sections before outlining how the current analysis could be used to structure localized analyses of expert knowledge and longitudinal data.

### 1.1. Challenges for Analysing Cascading Disaster Linkages

Huggins et al. [12] highlighted the potential for using localized, longitudinal data to study transitions from one disaster state to another. However, large and well-structured sets of relevant data are often not available for analysis. Kar-Purkayastha, Clarke, and Murray [13], and Huggins et al. [12] have outlined how open-access disaster impact databases typically lack important chronological, geographic, and other details. Associated challenges can be worsened by government agencies who are reluctant to allow researchers to access more detailed disaster impact data at a national scale [14]. Even where data is available, standardized impact assessment protocols often do not address the infrastructural impacts of meteorological hazards [15]. Other protocols require detailed analysis that is not usually feasible within many disaster-affected contexts [16].

All these challenges are exacerbated by rapidly changing urban development. Atta-ur-Rahman, Nawaz Khan, Collins, and Qazi [14] outlined how hazardous urban development in landslide-prone areas of Pakistan has been accelerating over time. Many other disaster-prone areas are also developing so rapidly that larger sets of longitudinal data do not apply to current urban footprints. The rapidly developing city of Shenzhen provides one example from within China’s Pearl River Delta. According to Swiss Re [17], this Delta is more heavily prone to storms, storm surge, and riverine flooding than any other metropolitan area in the world. It appears that the situation was not always so problematic because Shenzhen was formerly limited to the scale of a fishing town, prior to rapid development starting in the 1980s. Its urban footprint and potentially exposed population have since grown to a resident population of over 13 million people.

Issues concerning the structure, detail, and relevance of statistical hazard data mean it is often impossible to determine the base rate frequencies required for analysis such as the Bayesian Event Tree methods developed by Marzocchi, Sandri, and Selva [18]. However, these frequencies are not strictly required for predictive models based on the opinions of experienced and suitably qualified experts [19]. Relevant approaches to developing numerical models of potentially cascading disasters are exemplified by the combination of Cross Impact Analysis with Interpretive Structural Modelling (CIA-ISM), by Ramirez de la Huerga, Bañuls Silvera and Turoff [19]. Their method produces structural models of cascading disaster progressions by gathering, iterating, and then combining expert likelihood ratings, without using base rate frequency data.

Of course, no one analytical approach provides a panacea for the challenges of analyzing cascading disaster risk. Despite the many types of events that could be involved, Ramirez de la Huerga et al. [19] caution against adding too many triggers and impact parameters to the CIA-ISM process. This is because each parameter has a substantial effect on the number of expert ratings required. The importance of selecting the right set of initial rating parameters was demonstrated by Ramirez de la Huerga et al. [19] by reminding readers that the number of pathways requiring ratings is equivalent to N × 2^n−1^. This exponential relationship between parameters (N) and ratings required constrains the number of triggers and impacts that could be thoroughly considered by busy experts with limited time available.

### 1.2. Cascading Disaster Models Derived from Literature Reviews

Where appropriate data and expertise are available, wide-ranging literature reviews can help to constrain large sets of numerical parameters. Rather than providing an exhaustive list of possible triggers and impacts, they can refine analysis towards a more compact set of initial parameters that are well known to trigger one another. As outlined above, this is particularly important for expert-rating methods such as CIA-ISM [19]. Following the rationale and example provided by Mignan et al. [20], parameters could then be added or eliminated by experts, to reflect their professional knowledge of a particular context, or of a more generic set of mechanisms.

Among other examples, previous reviews of cascading disaster literature have resulted in a generalized model of freezing rain consequences by Schauwecker et al. [21], and a multi-hazard model constructed by Kumasaki, King, Arai, & Yang [22]. Schauwecker et al. [21] generalized from the basis of a single, freezing rain event in Slovenia. This meant that, although they also referred to a broader range of relevant cases, the context and particulars of their initial case resulted in a relatively deterministic pathway model, i.e., one that largely flowed from one determined consequence to another. Although this model included 17 different types of hazard events, only five of those event types could trigger two or more additional cascading pathways.

Kumasaki et al. [22] reviewed a much wider range of cases. They used their review of relevant documents to produce a much more exhaustive model of cascading pathways between documented natural hazard events that had occurred in Japan. The resulting model was also strengthened through specifying mechanisms for each of the cascading linkages. However, only 7 of 23 hazard types specified by Kumasaki et al. [22] branched into two or more further consequences. The specificity of these linkages may have been due to the particular geographic context of Japan, and relevant constraints on documenting the cases in question.

The specific scopes of Kumasaki et al. [22] and Schauwecker et al. [21] have nonetheless led to coherent and easily interpreted models of cascading disaster linkages. Their research outcomes could be compared to highly coherent scenario trees generated by Marzocchi et al. [18] and by Neri, Le Cozannet, Thierry, Bignami, and Ruch [23]. The main practical difficulty is that the compact coherence of these models is not so readily generalizable to a fuller range of geographical contexts and cascading hazards.

**Figure 1:**
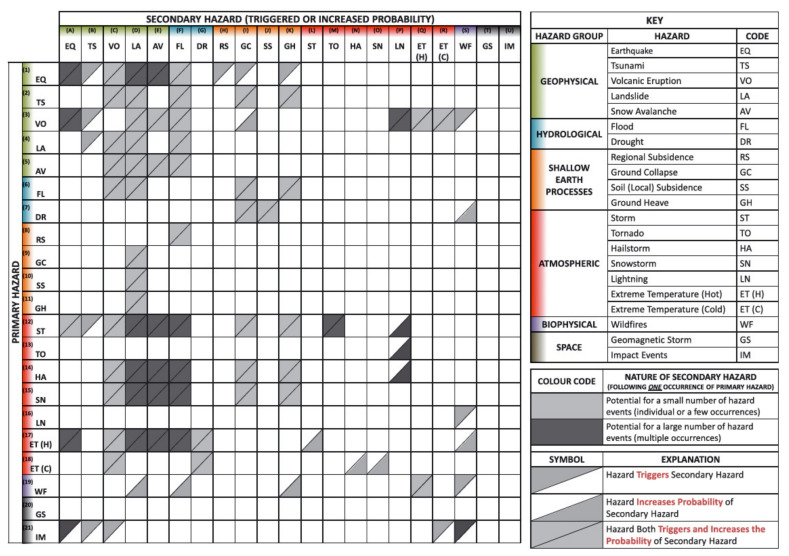
Identification of hazard interactions. Reproduced from “Reviewing and visualizing the interactions of natural hazards” by J. C. Gill and B. D. Malamud, 2014, Reviews of Geophysics, 52, p. 14. Copyright 2014 by the authors. Reproduced under the Creative Commons Attribution license 4.0.

Matrix models, like the one shown in Figure 1, provide a much less deterministic approach to the difficulties of predicting potentially cascading disasters because they highlight how several secondary hazards can be triggered by each event type.

This approach to defining multi-hazard linkages was exemplified by the Gill and Malamud [2], the authors of Figure 1, who systematically reviewed a wide range of case studies published in white and grey literature. Their review was summarized by this matrix of linkages from a set of 21 *primary* natural hazard triggers, listed vertically, and 21 types of *secondary* hazard events, listed horizontally. Grey triangles indicate a triggering or amplifying effect from a primary to a secondary hazard, resulting in a fairly exhaustive summary of which natural hazard types have historically triggered and/or worsened each other. Comparable matrices of inter-hazard linkages have also been produced by Tarvainen, Jarva, and Greiving [24], Kappes, Keiler, von Elverfeldt, and Glade [25], and by Mignan et al. [20].

## 2. Methods

As also exemplified by Gill and Malamud [2], the current methods were designed to fit the systematic literature review criteria from Boaz, Ashby, and Young [26]. These criteria require that a review: 1. Uses protocols to guide the process, 2. Is focused on a particular question, 3. Appraises the quality of the research, 4. Identifies as much of the relevant research as possible, 5. Synthesizes the research findings, 6. Aims to be as objective as possible, and 7. Is updated in order to remain relevant. The methods used to meet each one of these criteria are outlined in Table 1.

**Figure 2:**
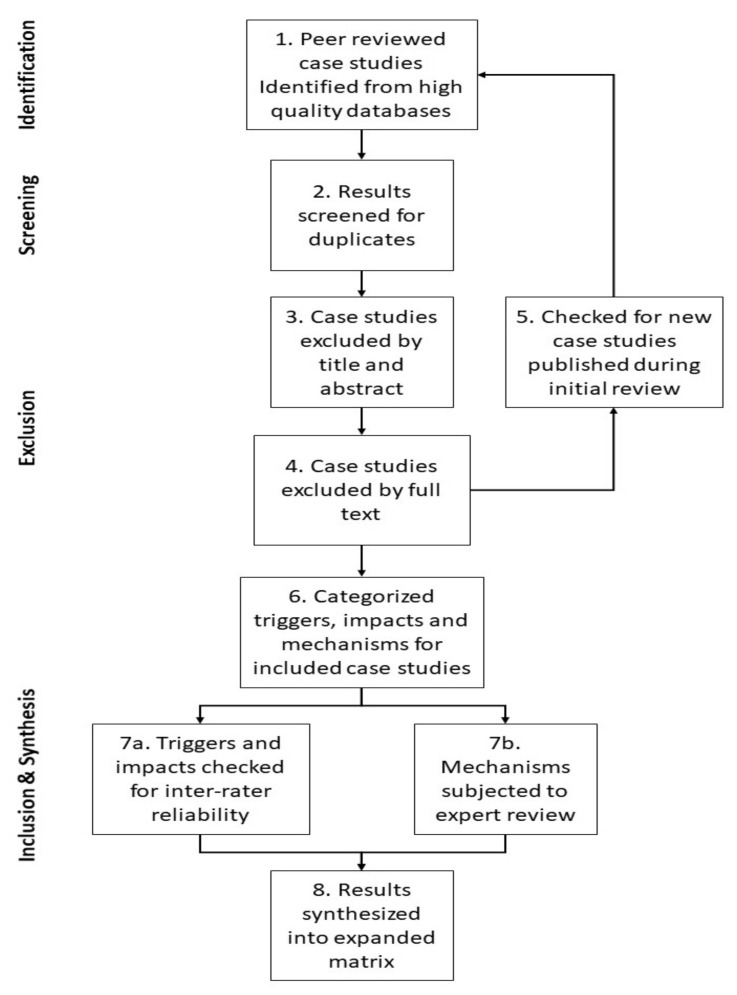
Overall method framework.

Figure 2 summarizes the overall process used to conduct the current literature review. Identification, screening, eligibility, and inclusion processes were incorporated from the standard PRISMA [27] protocol. Search results were generated by searching journal article texts for the natural hazards listed above, their common synonyms, and the terms “infrastructure” and “case study”.

Initial screening excluded all titles and abstracts that did not indicate at least one ground collapse, flood, landslide, storm, storm surge, or tornado case study. Titles and abstracts that did not indicate infrastructure impacts were also excluded. Eligible article texts outlined at least one relevant natural hazard event, and at least one infrastructural impact triggered by those events. Eligible texts also specified mechanisms explaining how each infrastructural impact was triggered.

Subsequent, qualitative synthesis used a set of established definitions, as outlined below, to categorize the rain-related triggers documented by each case study. A set of more generic terms were used to define the infrastructural impacts of these triggering events, as also outlined below. Trigger and impact categorizations were tested for inter-rater reliability, using a random sample of case study literature. Mechanisms linking triggers to secondary impacts were also categorized at this stage. Mechanism categories initially matched the original case study literature as closely as possible. They were then subjected to expert review, before being refined and included as part of the current results.

All reliable trigger-impact results matched with a valid mechanism were added to a selective, and slightly modified, version of the Gill and Malamud [2] matrix which is shown in Section 3 of the current paper. Impact magnitudes, scales, and durations were also recorded during this process. However, as shown in Table A1 (Appendix A), these data were not consistent enough for a more quantitative synthesis.

### Definitions

For consistency with the original Gill and Malamud matrix [2] (p. 11) of triggers and impacts, the same definitions were used to categorize rain-related natural hazard triggers:

*Avalanche*: The downslope displacement of surface materials (predominantly ice and snow) under gravitational forces.
*Ground Collapse*: Rapid, downward vertical movement of the ground surface into a void.
*Ground Heave*: The sudden or gradual, upward vertical movement of the ground surface.
*Landslide*: The downslope displacement of surface materials (predominantly rock and soil) under gravitational forces.
*Flood*: The inundation of typically dry land with water.
*Storm*: A significant perturbation of the atmospheric system, often involving heavy precipitation and violent winds.
*Tornado*: A violently rotating column of air pendant (normally) from a cumulonimbus cloud and in contact with the surface of the Earth.

Gill and Malamud [2] originally included *storm surge*, the landward movement of seawater resulting from a combination of heavy ocean-bound rainfall and tidal undulations, as a type of flood. This hazard was given its own category for the current research, to recognize the grave impacts of this increasingly common hazard. Frozen rain events, including hail, were excluded from the current analysis due to substantial differences between these types of hazards and more generic (liquid) rain-related triggers outlined by Schauwecker et al. [21]. Furthermore, and as shown in Figure 1, frozen rain events are not commonly triggered by liquid rainfall, being the focus of the current research.

Infrastructural impacts were not so difficult to define. This is because most people in the modern world are reliant on a broad range of infrastructures, as they go about their daily lives. Most people are also familiar with the failure of these infrastructure types. The following, relatively simplistic, definitions were therefore used to categorize impacted infrastructure:

*Agriculture*: Land developed for farming crops or livestock. Effectively critical for subsidence communities or settings characterized by low food security.
*Buildings*: Any private or public building that does not form part of other infrastructure categories.
*Electricity*: Stationary structures built for the generation and supply of electricity.
*Oil & Gas*: Stationary structures developed for the collection, refinement, and supply of oil or gas.
*Railway*: Stationary structures built for the transit of trains across the land, and bridges built for the transit of trains.
*Roads*: Stationary structures built for the transit of motor vehicles across the land, and bridges built for motor vehicle transit.
*Telecommunications*: Stationary structures built for the transmission of communications, including wired and mobile telephones.
*Water Supply*: Stationary structures developed to supply potable water for consumption.

## 3. Results

**Figure 3:**
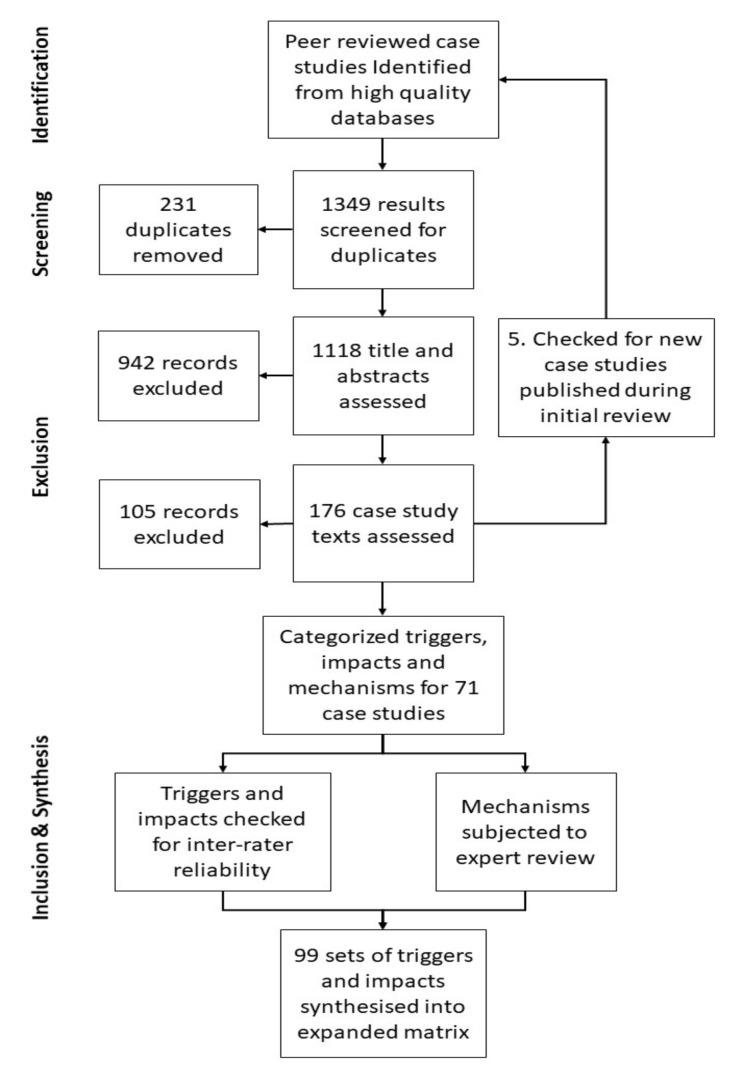
Progression through the systematic literature review protocol.

Figure 3 provides a standard PRISMA-based summary of how literature identification, screening, eligibility, and inclusion progressed from an initial set of 934 search results from the Web of Science Core Collection and 415 from the Scopus database. Once duplicates had been removed, a very large number of case study articles were excluded due to plainly irrelevant titles and abstracts. One hundred and five article texts were then excluded for failing to meet all criteria outlined in Section 2. Table 2 lists events and locations addressed by the 71 case study articles that were retained for synthesis.

Labels were assigned to each case of infrastructural failure outlined in retained article texts, using qualitative coding. During coding, it became apparent that ground heave is commonly recorded as a mechanism linking certain events to infrastructure damage, rather than being recorded as a discrete hazard. This helped explain the lack of articles outlining other mechanisms linking this hydro-geological process to infrastructure damage. There was only one article detailing relevant avalanche impacts, so this type of trigger was subsumed within a broadened landslide category. There were no articles clearly outlining applicable tornado hazard events, although relevant dynamics may have been subsumed within case studies of storm events.

Inter-rater reliability testing for natural hazard trigger and infrastructural impact codes was applied to a random stratified sample from the first 30 articles that had been analyzed. This included a total of 10 different articles, concerning 22 different impact occurrences. Coding instructions were improved until the analysis was 86% consistent between the different researchers. The resulting set of 71 articles concerned 99 cases of specific natural hazards triggering infrastructural impacts. These cases had occurred in 37 different countries and had involved a sum of 24 different mechanisms. Table 3 lists each mechanism identified while coding triggers and impacts, and then refined to reflect expert feedback.

**Figure 4:**
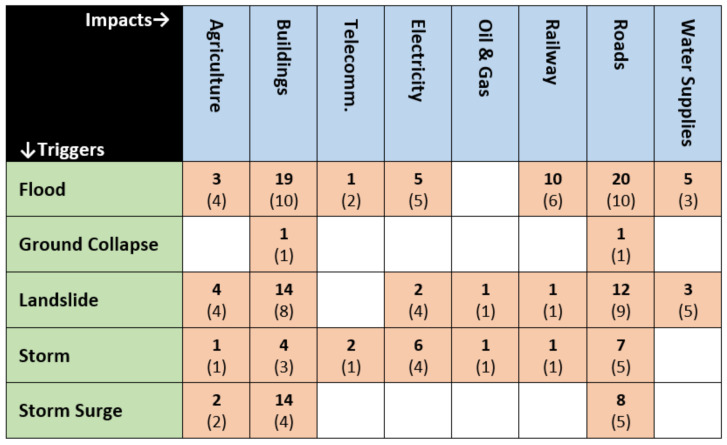
Matrix of natural hazard triggers and infrastructural impacts showing the number of cases in bold and the number of mechanisms in brackets.

Figure 4 combines the mechanisms shown in Table 3 with event frequencies, to display the validated linkages documented by eligible case study literature.

The bold numbers in each block indicate the total number of events where this linkage was well-documented by an eligible case study. The number of relevant mechanisms documented by the same literature is shown in brackets and plain type. There was often more than one mechanism involved in each event. This led to mechanism scores that are higher than event scores for some trigger-impact linkages.

**Figure 5:**
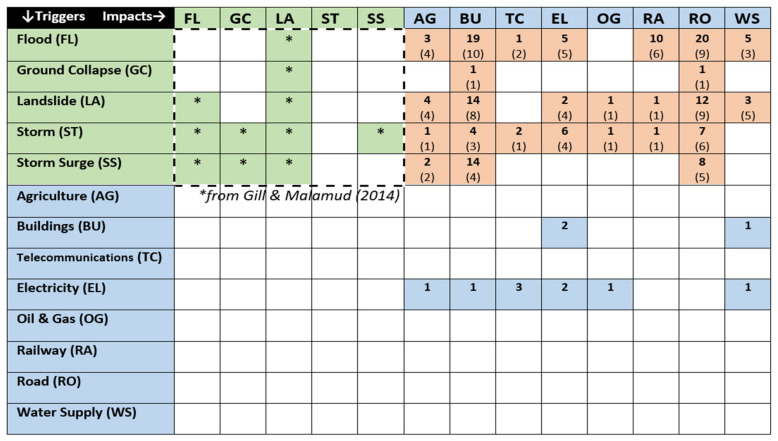
Matrix of triggers and impacts showing the number of cases in bold and the number of mechanisms in brackets.

The matrix shown in Figure 5 adds linkages from Figure 4 to rain-related triggers and impacts identified by Gill and Malamud [2]. Linkages between the latter set are marked with an asterisk. Linkages from natural hazards to natural hazards are shown in green, and linkages from natural hazards to infrastructural impacts are colored brown. The current matrix also includes infrastructure to infrastructure linkages, which were identified during the current review and have been colored blue.

The current literature review also identified 149 infrastructural impact magnitudes or scales, and 55 failure durations. However, substantially variable data formats and measurement units, combined with a very low statistical sample, meant that these more in-depth review data were not suitable for standard meta-analysis methods. There were comparable issues with the way impact magnitudes had been recorded, or not recorded, in the case studies being reviewed. Although this meant that the analysis of impact magnitudes, scales, and duration data was beyond the scope of the current research, a table summarizing raw data is provided in Appendix A.

## 4. Discussion

A comparable literature review of hurricane-related impacts on health infrastructure and non-communicable diseases by Ryan et al. [28], fully reviewed a sum of 19 relevant articles. The Gill and Malamud [2] review included a much larger total of over 200 cases. However, the latter review included a much wider scope and less restrictive inclusion criteria. The current set of 99 event cases is positioned in between each of these literature review antecedents, as is the current research scope.

The lack of a documented link between storm surge and power outages reflects conclusions from prior research. Tonn et al. [29] compared longitudinal relationships between various hurricane-related hazards and critical infrastructure impacts but found that storm surge did not have a substantial effect on power outages. They concluded that wind and precipitation rates had a much stronger relationship with electrical infrastructure failure. By contrast, flooding impacts account for a substantial proportion of the current linkage matrix shown in Figure 5. This echoes findings from other research, which have highlighted the disproportionate frequency and consequences of flooding disasters compared to other types of natural hazard events. According to an overview of the global Emergency Events Database (EM-DAT) by Cuñado and Ferreira [30] (p. 1), “Floods are the most common natural disaster accounting for 40 percent of all natural disasters between 1985 and 2009”. Together with storms, flooding accounted for 67 percent of losses recorded over the same period [30].

As outlined in Section 1 and Section 2, the current literature review does not provide a definitive list of all hazard linkages that have constituted cascading disasters. The current research was focused on events triggered by extreme rainfall and limited to case studies published in the English language. Even within these limitations, many relevant linkages would have been triggered by non-disastrous hazard events, outside the scope of generally disaster-focused case studies. Furthermore, the current literature review does not address how infrastructural impacts can amplify the impacts of natural hazard events and obstruct responding agencies [3], leading to highly complex disaster management scenarios. Caution is therefore required, to avoid over-interpreting the significance of the current results, and to remain mindful of how difficult it is to reliably predict the outcomes of complex interactions between diverse hazards, scales, and relevant social dynamics. As outlined in the Global Assessment Report on Disaster Risk Reduction [4], resulting disaster processes and impacts continue to surprise disaster management researchers and practitioners alike.

**Figure 6:**
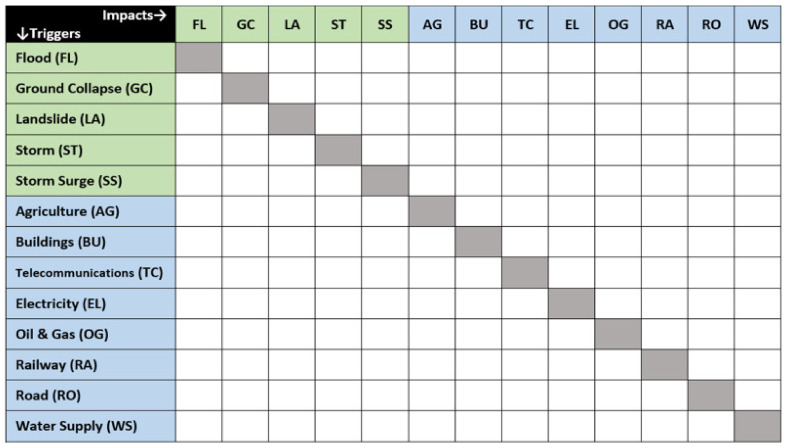
Matrix showing values for expert rating as blank white blocks.

The type of matrix shown in Figure 5 can nonetheless be used to reduce initial CIA-ISM or other Delphi-type parameters into a more workably compact set of expert rated values. As shown in Figure 6, an expert rating matrix derived from Figure 5 can then be used to efficiently analyze the likelihoods of rain-related disaster linkages. Experts would simply be asked to assign probabilities to each of the blank white rectangles shown in Figure 6. This is how the current extension of the Gill and Malamud [2] matrix could be used to create more detailed scenarios of rain-related disaster cascades, including infrastructural impacts.

Numerical values from Figure 5 can provide approximate base-rate linkage frequencies, between natural hazard triggers and infrastructural impacts. The same applies to approximations from the original matrices produced by Gill and Malamud [2]. Where permitted by an expert rating protocol, experts could be prompted to consider both sets of values. This would help mitigate a perceptual bias called the *base-rate fallacy*, where individuals tend to inflate the likelihood of recent disaster linkages, by ensuring that each expert considers how relatively infrequently those linkages occur [12].

**Figure 7:**
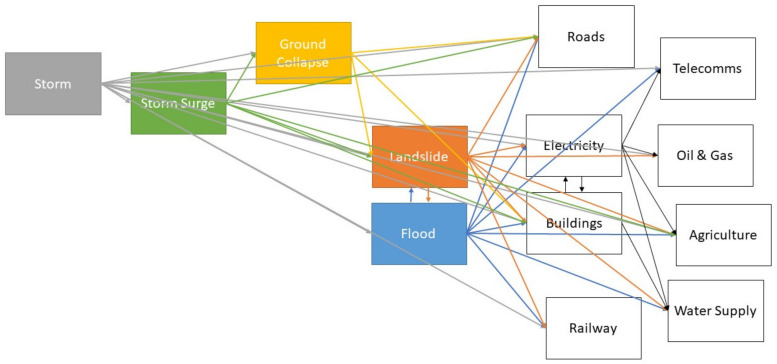
Network model framework summarizing literature review results.

The literature review results summarized in Figure 5 can also be used to shape network-orientated analyses based on empirical data. In principle, this would involve assigning values to the type of linkages shown in Figure 7. Given appropriate data, relevant approaches to network analysis could provide a data-driven alternative to the type of scenario model generated by Schauwecker et al. [21]. Even without assigning values to the links shown in Figure 7, the current qualitative synthesis suggests that landslides and floods are particularly central nodes. However, a network analysis of quantitatively consistent data would produce a much more robust conclusion.

Where possible, subsequent expert-rating protocols or network frameworks informed by the current research should still be subject to piloting and adjustment for specific geographic areas. This can include local expert feedback on possible alterations and additions, to avoid excluding salient linkages. The importance of these expert modifications was illustrated by Mignan et al. [20], who developed an expansive set of potential multi-hazard linkages through consulting with high school teachers who were specialized in natural sciences. The participants made several additions to hazard linkages that had been previously documented. Drawing on their own expert knowledge, Mignan et al. [20] concluded that each of these additional linkages was reasonable and that they could realistically occur.

## 5. Conclusions

Cascading disasters progress from one type of hazard to others, with consequences that are often devastating [3]. Rain-related cascading disasters are particularly frequent in many parts of the world, leading to repeatedly catastrophic impacts. These types of disasters are likely to become even more frequent due to climate change [7], and accelerating development in areas prone to relevant hazards [5,9].

Infrastructural impacts often result from natural hazard triggers. These types of impacts can form a particularly catastrophic and even amplifying aspect of cascading disaster scenarios [6]. However, to the best of the authors’ current knowledge, cascading linkages from rain-related natural hazards to infrastructural impacts have not previously been addressed by systematic case study reviews. To address this gap in scientific knowledge, the current literature review focused on mechanisms leading to infrastructural impacts in particular. This is how the current results have defined much of what is known about linkages between rain-related triggers and infrastructural impacts amounting to cascading disaster risk. A range of mechanisms constituting these linkages have also been identified by the current research.

A sum of 71 articles, concerning 99 case studies of rain-related disasters, were reviewed using a systematic literature review protocol. This was restricted to case studies detailing the mechanisms that have led to infrastructural impacts, and which had been indexed in high-quality academic journal databases. Twenty-five distinct mechanisms were identified as a result. These were combined with linkages previously identified through a systematic case study review by Gill and Malamud [2], to form a matrix running between five different natural hazards and eight types of infrastructural impacts.

The resulting matrix, shown in Figure 6, is principally designed for structuring expert rating analyses of rain-related cascading disaster scenarios. It can be used for Delphi-based, cross-impact analysis [19,31], as an initial set of rating parameters which reduce the time and attention required from expert raters. Base-rate approximations included in this matrix can be added to a range of approximations from Gill and Malamud [2], to mitigate known biases. The same matrix, or the graphic shown in Figure 7, could also be used to identify key parameters in longitudinal analyses of cascading rain-related hazard events. These key parameters could help to collect and structure available data, including social media. This is one way that the current results can be used to transparently structure a range of quantitative analyses, including analyses leveraging artificial intelligence.

## Figures and Tables

**Table 1:** Review criteria applied to the current research.

Criteria	Application
Follows a Protocol	Followed steps outlined in the Preferred Reporting Items for Systematic Reviews and Meta-Analyses (PRISMA) protocol [27]: Identification, Screening, Eligibility, Inclusion.
Answers a Research Question	Answered: 1. What are the infrastructural impacts resulting from rain-related hazards? 2. What are the mechanisms explaining how each impact was caused?
Appraises Research Quality	Reviewed academic journal articles, subject to relatively standardized peer review processes. All identified mechanisms subject to review from a disaster resilience and civil engineering expert.
Addresses as Much Research as Possible	Drew on more than 22,800 publications covered by Scopus and 21,177 covered by the Web of Science Core Collection.
Synthesizes Research Findings	Findings synthesized into a selective extension of a pre-existing matrix from Gill and Malamud [2].
As Objective as Possible	Key parts of coding framework subject to inter-rater reliability testing.
Update in Order to Remain Relevant	All database searches updated within two weeks of initial review.

**Table 2:** Events and Locations Addressed by Eligible Case Studies.

Year	Event	Location	Country
Not dated (n.d.)	Not named	Flanders	Belgium
n.d.	Not named	Northeast Area	USA
1831	Not named	Avarua	Cook Islands
1871	Cartago Floods	Cartago City	Costa Rica
1935	Not named	Avarua	Cook Islands
1946	Not named	Ngatangiia	Cook Islands
1962	Not named	Mid-Atlantic Coast	USA
1967	Not named	Avarua	Cook Islands
1974	Not named	ltmündener Wand	Germany
1985	Not named	Tibet	China
1987	Cyclone Sally	Avarua	Cook Islands
1987	Not named	Martell Valley	Italy
1988	Not named	Midui	China
1993	Not named	Zêzere Valley	Portugal
	Not named	Sirwolte	Switzerland
1994	Phojal Nalla Flood	Kullu District	India
1995	Not named	Vorarlberg	Austria
1997	Bugobero Village Landslide	Bugobero	Uganda
1999	Not named	New York City	USA
	Not named	Teziutlán	Mexico
	Odisha Super Typhoon	Odisha	India
2001	Tropical Storm Allison	Texas	USA
2002	Not named	Eilenberg	Germany
2003	Not named	New York City	USA
2004	Cyclone Heta	Avarua	Cook Islands
	Not named	Hua-Qing Highway	China
	Not named	Northern Apennines	Italy
	Sextas Landslide	Tena Valley	Spain
	Typhoon No. 23	Kansai	Japan
2005	Cyclone Meena	Avarua	Cook Islands
	Cyclone Nancy	Matavera	Cook Islands
		Ngatangiia Harbour	Cook Islands
	Hurricane Katrina	Gulf Coast	USA
		New Orleans	USA
	Not named	Apulia	Italy
	Not named	Zêzere Valley	Portugal
	Not named	Carlisle	UK
2006	March River Flood	March River	Austria
2007	Cyclone Sidr	Sarankhola Upazi	Bangladesh
	Not named	Altay	China
2008	Not named	Solent	UK
	Sextas Landslide	Tena Valley	Spain
2009	La Selva Landslide	Tena Valley	Spain
	Not named	Tianmo	China
2009 to 2011	Not named	Calabria	Italy
2010	Central Indus Basin Floods	Muzaffargarh	Pakistan
	Not named	Calabria	Italy
	Not named	Gimigliano	Italy
	Not named	San Fratello	Italy
2011	Not named	Chia	Colombia
	Not named	Syracuse	USA
	Typhoon Roke	Tokai, Japan	
2012	Hurricane Sandy	Connecticut	USA
		New Jersey	USA
		New York	USA
2012	Not named	Beijing	China
	Not named	Haitong	China
	Not named	Xiqu	China
	Not named	South-West Dieppe	France
	Superstorm Sandy	New York	USA
2013	Central Europe Floods	Not specified	Germany
	Colorado Floods	Boulder County	USA
	Cyclone Phailin	Odisha	India
	Not named	Not specified	Austria
	Not named	Peace River	Canada
	Not named	Garhwal Himalaya	India
	Not named	Piedmont	Italy
	Not named	Far East Russia	Russia
	Not named	Norrala	Sweden
	Typhoon Haiyan	Tacloban City	Philippines
2014	Madeira River Floods	Madeira River	Brazil
	Not named	Acre State	Brazil
	Not named	Outer Carpathian	Poland
	Not named	Loch Insh	Scotland
	Not named	Not specified	Slovenia
	Not named	Värmland	Sweden
	Not named	Västra Götaland	Sweden
2015	Hurricane Patricia	Colima	Mexico
	Not named	Rest and be Thankful	Scotland
	Tropical Storm Erika	Not Specified	Dominica
2016	Hurricane Matthew	Princeville	USA
2017	Hurricane Harvey	Houston	USA
	Hurricane Irma	Florida	USA
	Not named	Jushui Basin	Japan

**Table 3:** Mechanisms by natural hazard trigger and infrastructural impact type.

Trigger	Impacted Infrastructure	Mechanisms
Flood	Agriculture	Blockage, Debris Transport, Erosion, Inundation
	Buildings	Burying, Contamination, Debris Transport, Destabilization, Erosion, Force, Impact, Incision, Inundation, Scour
	Telecommunications	Impact, Scour
	Electricity	Burying, Debris Transport, Erosion, Force, Inundation
	Railway	Burying, Erosion, Force, Inundation, Subsidence, Undermining
	Roads	Burying, Debris Transport, Erosion, Force, Impact, Incision, Inundation, Scour, Sediment Transport, Subsidence
	Water Supply	Contamination, Debris Transport, Inundation
Ground Collapse	Buildings	Subsidence
	Roads	Subsidence
Landslide	Agriculture	Burying, Erosion, Displacement, Subsidence
	Buildings	Burying, Debris Transport, Erosion, Force, Impact, Settling, Subsidence, Translation
	Electricity	Displacement, Erosion, Force, Subsidence
	Oil & Gas	Displacement
	Railway	Sediment Transport
	Roads	Blockage, Burying, Debris Transport, Displacement, Erosion, Impact, Sediment Transport, Subsidence, Translation
	Water Supply	Displacement, Erosion, Force, Subsidence, Translation
Storm	Agriculture	Inundation
	Buildings	Inundation, Mold, Wind
	Telecommunications	Wind
	Electricity	Lightning, Snow Load, Tree Fall, Wind
	Oil & Gas	Wind
	Railway	Wind
	Roads	Erosion, Ice, Inundation, Tree Fall, Wind
Storm Surge	Agriculture	Inundation, Salination
	Buildings	Debris Transport, Erosion, Impact, Inundation
	Roads	Debris Transport, Erosion, Inundation, Scour, Undermining

## Appendix A

**Table A1:** Review criteria applied to the current research.

Event Cases	Trigger	Magnitude	CI Type	Impacts	Impact Scale	Impact Duration
Central Indus Basin Floods, Muzaffargarh, Pakistan, July 2010	Flood	Approx. 1.04 ft/s peak discharge	Agriculture	Cotton, rice and sugarcane crops destroyed	106 ha	3 weeks
Unnamed Event, Garhwal Himalaya, India, June 2013	Flood	River gradient increase to 68 m/km	Agriculture	Destroyed	17 ha of farmland	Not specified
Unnamed Event, Garhwal Himalaya, India, June 2013	Flood	River level increase of >30 m	Agriculture	Destroyed	3.3 × 10^6^ km of farmland	Not specified
Madeira River Floods, Madeira River, Brazil, April 2014	Flood	20 m rise in river level, above normal level	Buildings	Damaged	0.65 km^2^ of urban area, containing 27 public buildings	Not specified
Unnamed Event, Garhwal Himalaya, India, June 2013	Flood	River level increase of approximately 32 m	Buildings	Destroyed	>10 shops, four houses, two hotels, one big temple, one large motor workshop	Not specified
Hurricane Harvey Houston, USA, August 2017	Flood	Not specified	Buildings	Hospital closed	1 hospital	4 days
Tropical Storm Allison, Texas, USA, June 2001	Flood	425 m^3^s 765 m^3^s flow rate	Buildings	Damaged	1 hospital	Not specified
Unnamed event, Zêzere Valley, Portugal, 1993	Flood	Not specified	Buildings	Damaged	1 hotel	Not specified
Unnamed event, Sirwolte, Switzerland, September 1993	Flood	150,000 m^3^ of water from glacier lake breach. 400 m^3^/s or 320 m^3^/s peak discharge	Buildings	Destroyed	1 house	Not specified
Unnamed event, New York City, USA, June 2003	Flood	Not specified	Buildings	Damaged	1 house	Not specified
Unnamed event, Altai, Russia, Autumn 2013	Flood	8,000,000 km^2^	Buildings	Damaged	12,643 houses, 402 social facilities	Not specified
Unnamed Event, Chia, Colombia, April–May 2011	Flood	100-year event	Buildings	Houses inundated	1455 urban plots	Not specified
Central Indus Basin Floods, Muzaffargarh, Pakistan, July 2010	Flood	Approx. 1.04 ft/s peak discharge	Buildings	Houses fully to partially damaged	1491 houses in flooded area, at a cost of USD 586,642 for replacement or repair	Not specified
Unnamed Event, Garhwal Himalaya, India, June 2013	Flood	River gradient increase to 68 m/km	Buildings	Buried	2.3 × 10^4^ m^2^ village	Not specified
Unnamed Event, Altay, China, Spring 2007	Flood	Covering 386.39 km^2^	Buildings	Damaged	2375 households and 6388 rooms	Not specified
Unnamed Event, Garhwal Himalaya, India, June 2013	Flood	River level increase of >30 m	Buildings	Destroyed	3 large hotels	Not specified
Unnamed event, New York City, USA, January 1999	Flood	76mm/h of rainfall	Buildings	Inundated to within 152.4 mm of ceilings	30 block residential area	Not specified
Unnamed event, Carlisle, UK, January 2005	Flood	Average depth of 1.79 m	Buildings	Damaged	322,950 m^2^	Not specified
Tropical Storm Allison, Texas, USA, June 2001	Flood	425 m^3^s 765 m^3^s flow rate	Buildings	Damaged	4 hospitals	Up to 5 weeks
Unnamed event, Eilenberg, Germany, August 2002	Flood	Average depth of 1.91 m	Buildings	Damaged	529,725 m^2^	Not specified
Tropical Storm Allison, Texas, USA, June 2001	Flood	425 m^3^s 765 m^3^s flow rate	Buildings	Damaged	6 hospitals	Up to 5 weeks
Unnamed event, Outer Carpathian, Poland, August 2014	Flood	2.5 above floodplain terrace, with flow of between 1.6 and 2.0 ms^−1^	Buildings	Damaged	70 farm buildings	Not specified
Unnamed event, Eilenburg, Germany, August 2002	Flood	3 m deep urban inundation	Buildings	Damaged	765 buildings	Not specified
Unnamed Event, Garhwal Himalaya, India, June 2013	Flood	Not specified	Buildings	Buried	Entire town	Not specified
Unnamed Event, Garhwal Himalaya, India, June 2013	Flood	River gradient increase to 243 m/km	Buildings	Destroyed	Entire village	Not specified
Unnamed Event, Garhwal Himalaya, India, June 2013	Flood	~2.09 × 106 m^3^ of debris flow	Buildings	Destroyed	Entire village	Not specified
Unnamed Event, Garhwal Himalaya, India, June 2013	Flood	River level increase of 50 m	Buildings	Destroyed	Entire village	Not specified
Unnamed Event, Garhwal Himalaya, India, June 2013	Flood	River level increase of 30–40 m	Buildings	Destroyed	Lower part of Govindghat village	Not specified
Unnamed event, Martell Valley, Italy, August 1987	Flood	300–500 m^3^ of water released from reservoir	Buildings	Houses, industrial and agricultural buildings damaged or demolished and swept away	Mainly affected three villages	Not specified
Cartago Floods, Cartago City, Costa Rica, October 1871	Flood	More than 2 m of debris flow, leaving up to 1 m of mud	Buildings	Damaged and destroyed	More than 120 houses	Not specified
Unnamed Event, Garhwal Himalaya, India, June 2013	Flood	Approx. 15–20 m rise in river level	Buildings	Destroyed	Various settlements	Not specified
Unnamed Event, Jushui Basin, Japan, July 2017	Flood	Mainly between 0 to 2 m deep	Buildings	Water-logged houses	Yellow Lake community	4 days
Martell Valley, Italy, August 1987	Flood	300–500 m^3^ of water released from reservoir	Communications	Significantly damaged	1 village	Not specified
Central Indus Basin Floods, Muzaffargarh, Pakistan, July 2010	Flood	Approx. 1.04 ft/s peak discharge	Electricity	Power poles damaged	30 power poles, at a cost of USD 50,000	Not specified
Tropical Storm Allison, Texas, USA, June 2001	Flood	425 m^3^s 765 m^3^s flow rate, causing up to 12 m of flooding	Electricity	Power cut	4 hospitals	Up to 4 days
Unnamed Event, Garhwal Himalaya, India, June 2013	Flood	River level increase of >30 m	Electricity	Destroyed	Hydropower plant	Not specified
Unnamed Event, Garhwal Himalaya, India, June 2013	Flood	Not specified	Electricity	Destroyed	Hydropower plant	Not specified
Unnamed Event, Garhwal Himalaya, India, June 2013	Flood	River level increase of approximately 32 m	Electricity	Filled up	1 hydropower plant	Not specified
Unnamed event, Martell Valley, Italy, August 1987	Flood	300–500 m^3^ of water released from reservoir	Electricity	Significantly damaged telephone network	1 village	Not specified
Unnamed Event, Garhwal Himalaya, India, June 2013	Flood	River gradient increase to 243 m/km	Electricity	Buried	Powerhouse	Not specified
Unnamed event, March River Flood, Austria, 2006	Flood	Average flow of 108 m^3^ s^−1^, peak flow of 1400 m^3^ s^−1^	Railway	Damaged	>10 km of track	Not specified
Unnamed Event, Austria, June 2013	Flood	From up to 300 mm or rainfall, leading to a more than 100-year discharge rate	Railway	Destroyed	1 bridge	Not specified
Unnamed Event, Vorarlberg, Austria, 1995	Flood	Not specified	Railway	Derailment causing 3 deaths and 17 severe injuries	1 train	Not specified
Central Europe Floods, Germany, 2013	Flood	Not specified	Railway	Closed and interrupted	75 track sections	Service disruptions of up to 5 months
Unnamed Event, Norrala, Sweden, August 2013	Flood	90 mm of rain in 3 h	Railway	Tunnel blocked	1 4 km tunnel	1 day
Unnamed event, New York City, USA, June 2003	Flood	Not specified	Railway	Closed	Several subway lines	Not specified
Unnamed event, Västra Götaland, Sweden, August 2014	Flood	Not specified	Railway	Embankment damaged	Up to 20 mm of embankment at 2 sites	Not specified
Unnamed Event, Xiqu, China, June 2012	Flood	100 m length and 210 m of debris flow	Roads	Destroyed highway section	>200 m of highway pavement	Not specified
Unnamed event, Värmland, Sweden, August 2014	Flood	From maximum 87 mm/day rainfall	Roads	Closed	1 highway	Not specified
Unnamed Event, Altay, China, Spring 2007	Flood	Covering 386.39 km^2^	Roads	Damaged	102 km	Not specified
Unnamed Event, Haitong, China, June 2012	Flood	Not specified	Roads	Barrier lake formed	160 m of subgrade	Not specified
Unnamed Event, Tianmo, China, July 2009	Flood	Not specified	Roads	Sub-grade destroyed	1 km	Not specified
Unnamed event, New York City, USA, June 2003	Flood	Not specified	Roads	Blocked by up to 3 m of water	2 intersections	Not specified
Unnamed event, Acre State, Brazil, 2014	Flood	Not specified	Roads	Highway blocked	22 municipalities	60 days
Unnamed event, Piedmont, Italy, April–June 2013	Flood	20 debris flows	Roads	Road wall collapse, jammed bridges, other damage	3700 km^2^ area withabout 420,000 inhabitants	Not specified
Unnamed Event, Garhwal Himalaya, India, June 2013	Flood	River level increase of >30 m	Roads	Destroyed	400 m	Not specified
Unnamed event, Russian Far East, Russia, Autumn 2013	Flood	8,000,000 km^2^	Roads	Flooded and damaged	4346 km	8 weeks
Unnamed Event, Garhwal Himalaya, India, June 2013	Flood	~15–20 m rise in river level	Roads	Blocked	4 m diameter tunnel	Not specified
Unnamed Event, Xiqu, China, June 2012	Flood	From barrier lake with average width of 60 m and average depth of 5–6 m	Roads	Destroyed highway section	500 m of highway pavement	Not specified
Unnamed Event, Garhwal Himalaya, India, June 2013	Flood	River level increase of approximately 30 m	Roads	Destroyed	5 km	Not specified
Unnamed Event, Garhwal Himalaya, India, June 2013	Flood	River gradient increase to 243 m/km	Roads	Destroyed	80 km	Not specified
Unnamed Event, Xiqu, China, June 2012	Flood	22 simultaneous debris flows	Roads	Interrupted Sichuen-Tibet Highway, with 100 vehicles and at least 300 people trapped	Eight sections of highway	10 days until highway restored
Tropical Storm Erika, Dominica, August 2015	Flood	Up to 400 mm of rain within four hours	Roads	Blocked	Main road	At least 3 years
Unnamed event, Zêzere Valley, Portugal, October 2005	Flood	34 debris flows	Roads	Closed	National Highway	Not specified
Unnamed event, Västra Götaland, Sweden, August 2014	Flood	Not specified	Roads	Bridge destroyed	One 5 m span bridge	Not specified
Hurricane Harvey Houston, USA, August 2017	Flood	Not specified	Roads	Blocked	One highway, 200 road sections	4 days
Martell Valley, Italy, August 1987	Flood	300–500 m^3^ of water released from reservoir	Roads	Destroyed or buried	One village	Not specified
Unnamed Event, Calabria, Italy, 2009 to 2011	Flood	Not specified	Roads	Interrupted transit	Several hamlets isolated	Not specified
Unnamed event, New York City, USA, June 2003	Flood	Not specified	Roads	Closed	Several roads	Not specified
Unnamed event, Syracuse, USA, April 2011	Flood	Not specified	Roads	Closed	Several roads	Several days
Unnamed Event, Tibet, China, June 1985	Flood	Not specified	Roads	Closed	Sichuan-Tibet Highway	7 months
Unnamed Event, Midui, China, July 1988	Flood	Not specified	Roads	Interrupted	Sichuan-Tibet Highway	More than 6 months
Unnamed event, New York City, USA, January 1999	Flood	76 mm/h of rainfall	Roads	Inundated	Three neighbourhoo-ds	Not specified
Colorado Floods, Boulder County, USA, September 2013	Flood	Resulting from more than 500 mm of rain	Roads	Blocked roads	Throughout City of Longmont	Not specified
Unnamed event, Västra Götaland, Sweden, August 2014	Flood	Not specified	Roads	Closed	Two roads	Not specified
Tropical Storm Allison, Houston, USA, June 2001	Flood	425 m^3^s 765 m^3^s flow rate	Water	Disrupted	1 hospital	Not specified
Central Indus Basin Floods, Muzaffargarh, Pakistan, July 2010	Flood	Approx 1.04 ft/s peak discharge exceeding capacity of local barrages and dams. Century worst flood event, killing more than 1900 people	Water	Damaged canal network	114 km of irrigation network	Not specified
Madeira River Floods, Madeira River, Brazil, April 2014	Flood	20 m rise in river level, above normal level	Water	Contaminated drinking water	15% of municipal population	Not specified
Hurricane Matthew, Princeville, USA, October 2016	Flood	Not specified	Water	Water treatment failed	City-wide	Not specified
Unnamed event, Martell Valley, Italy, August 1987	Flood	300–500 m^3^ of water released from reservoir	Water	Significantly damaged.	One village	Not specified
Unnamed event, Apulia, Italy, October 2005	Flooding	6.3 m impoundment	Railway	Damaged	1 section of rail embankment	Not specified
Unnamed event, South-West Dieppe, France, December 2012	Ground collapse	100,000 m^3^	Buildings	House on 40 m of cliff edge destroyed	1 house	Not specified
Unnamed event, Northern Apennines, Italy, April 2004	Landslide	100’s of shallow landslides	Agriculture	Damaged	Not specified	3 months
Unnamed events, Flanders, Belgium, n.d.	Landslide	Not specified	Agriculture	Damaged	Not specified	Not specified
Phojal Nalla Flood, Kullu District, India, August 1994	Landslide	Not specified	Agriculture	Arable land lost	Not specified	Not specified
Bugobero Village Landslide, Bugobero, Uganda, December 1997	Landslide	100,000 m^3^ moved 2.5 km	Agriculture	Destroyed plantations	Not specified	Not specified
Unnamed event, Calabria, Italy, February 2010	Landslide	Length of ~400 m, width of ~120 m, an area of ~4.8 ha, estimated volume of ~720,000 m^3^, mean slope gradient of ~17°, and 3 m scarp	Buildings	Destroyed and damaged	1 petrol station and a number of houses	Not specified
Sextas Landslide, Tena Valley, Spain, Summer 2004	Landslide	Not specified	Buildings	Damaged	1 ski-field chair lift	Not specified
Unnamed Event, San Fratello, Italy, February 2010	Landslide	8–10 m surface rupture, landslide 1.8 km long	Buildings	Severely damaged and destroyed buildings including a church and school	1 km^2^	Not specified
Typhoon No. 23, Kansai, Japan, October 2004	Landslide	230 m long, including 23 m high reinforced earth wall	Buildings	Damaged	1 warehouse	Not specified
Unnamed event, Teziutlán, Mexico, October 1999	Landslide	Not specified	Buildings	Buried	Part of a village	Not specified
Sextas Landslide, Tena Valley, Spain, June 2008	Landslide	420 m long, 100 wide, with 35 m scarp	Buildings	Damaged	Snow cannon infrastructure	Not specified
Unnamed event, Flanders, Belgium, n.d.	Landslide	Not specified	Electricity	Damaged	1 cable	Not specified
Central Europe Floods, Germany, 2013	Landslide	Not specified	Railway	Closed and interrupted	75 track sections	Service disruptions of up to 5 months
Unnamed event, Gimigliano, Italy, January 2010	Landslide	Not specified	Roads	Destabilised	1 bridge	Not specified
Hurricane Patricia, Colima, Mexico, October 2015	Landslide	Not specified	Roads	Bridge destroyed	1 bridge	Not specified
La Selva Landslide, Tena Valley, Spain, April 2009	Landslide	145 cm/year movement	Roads	Major damages	1 road	Not specified
Unnamed event, Calabria, Italy, February 2010	Landslide	Length of ~400 m, width of ~120 m, an area of ~4.8 ha, estimated volume of ~720,000 m^3^, mean slope gradient of ~17°, and 3 m scarp	Roads	Disrupted	1 road	Not specified
Unnamed Event, San Fratello, Italy, February 2010	Landslide	8–10 m surface rupture, landslide 1.8 km long	Roads	Destroyed	1 km^2^	Not specified
Unnamed event, Piedmont, Italy, April–June 2013	Landslide	300 landslides	Roads	Road wall collapse, jammed bridges, other damage	3700 km^2^ area withabout 420,000 inhabitants	Not specified
Unnamed Event, Rest and be Thankful, Scotland, December 2015	Landslide	100 m^3^ of earth movement	Roads	Barrier failed and slope instability, highway closed	Not specified	7 days
Unnamed Event, ltmündener Wand, Germany, Winter 1974	Landslide	Not specified	Roads	Highway blocked	On highway route	Not specified
Unnamed event, Peace River, Canada, May 2013	Landslide	Not specified	Roads	Closed	One highway	Several months
Unnamed Event, Calabria, Italy, 2009 to 2011	Landslide	Not specified	Roads	Interrupted transit	Several hamlets isolated	Not specified
Unnamed Event, San Fratello, Italy, February 2010	Landslide	8–10 m surface rupture, landslide 1.8 km long	Water	Damaged and destroyed drainpipes	1 km^2^	Not specified
Cyclone Sidr, Sarankhola Upazi, Bangladesh, November 2007	Storm	Category 4 cyclone, with average wind speed of 237 km/h	Agriculture	Cropland destroyed	0.65 million ha	Not specified
Cyclone Sidr, Sarankhola Upazi, Bangladesh, November 2007	Storm	Category 4 cyclone, with average wind speed of 237 km/h	Buildings	Houses destroyed	1.2 million	Not specified
Hurricane Sandy, Rockaway Peninsula, USA, October 2012	Storm	Not specified	Buildings	Damaged	16 of 46 primary health facilities	Not specified
Hurricane Sandy, Rockaway Peninsula, USA, October 2012	Storm	Not specified.	Buildings	Damaged	24 of 46 primary health facilities	Not specified
Hurricane Katrina, New Orleans, USA, August 2005	Storm	Not specified	Buildings	Inundated	80% of city	Not specified
Hurricane Irma, Florida, USA, September 2017	Storm	Category 4 hurricane, with winds up to 119 kp/h and rainfall of up to 550 mm within 96 hours	Buildings	Severely damaged or destroyed	Most houses in Florida Keys County	Not specified
Hurricane Katrina, Gulf Coast, USA, August 2005	Storm	Not specified	Communications	Damaged or collapsed	Entire Gulf Area	Not specified
Unnamed Event, Slovenia, January to February 2014	Storm	Freezing rain of up to 150 mm/hr	Electricity	Power cut	250,000 people	Not specified
Hurricane Irma, Florida, USA, September 2017	Storm	Category 4 hurricane, with winds up to 119 kph and rainfall of up to 550 mm within 96 hours	Electricity	Power cut	36% of Florida customers	10 days
Unnamed Event, Northeast United States, n.d.	Storm	Not specified	Electricity	22,700 MW of power supply interrupted	380,000 customers	Not specified
Hurricane Katrina, Gulf Coast, USA, August 2005	Storm	Not specified	Electricity	Damaged or collapsed	Entire Gulf Area	Not specified
Cyclone Phailin, Odisha, India, October 2013	Storm	Category 5 hurricane, with sustained wind speeds up to 215 km/h	Electricity	Power cut	North and West of state, 1,500 MW of electricity transmission lost	1 week
Cyclone Phailin, Odisha, India, October 2013	Storm	Category 5 hurricane, with sustained wind speeds up to 215 km/h	Electricity	Rural power cut	Not specified	1 month
Cyclone Phailin, Odisha, India, October 2013	Storm	Category 5 hurricane, with sustained wind speeds up to 215 km/h	Electricity	Urban power cut	Not specified	1 week
Hurricane Sandy, New Jersey and New York, USA, October 2012	Storm	Wind gusts >120 kp/h, Approximately 1770 km storm diameter	Electricity	Disrupted	Not specified	More than 1 week
Hurricane Sandy, Connecticut, USA, October 2012	Storm	Maximum wind speed of 16 m/s^−1^	Electricity	Power cut	Over 500,000 customers	Up to 9 days
Unnamed event, Hua-Qing Highway, China, 2004	Storm	Not specified	Roads	Disrupted	1 highway	Not specified
Unnamed Event, Loch Insh, Scotland, December 2014	Storm	Not specified	Roads	Embankment failed	20 m, with a 10 m vertical face	Not specified
Typhoon Roke, Tokai, Japan, September 2011	Storm	496 mm of rain, with intensities up to 78 mm/h	Roads	Blocked	333 locations	Not specified
Unnamed Event, Beijing, China, July 2012	Storm	From >460 mm of rain in under 24 hours	Roads	Blocked	63 roads	Not specified
Cyclone Sidr, Sarankhola Upazi, Bangladesh, November 2007	Storm	Category 4 cyclone, with average wind speed of 237 km/h	Roads	Roads and embankments destroyed or damaged	85% of region infrastructure	Not specified
Cyclone Sidr, Sarankhola Upazi, Bangladesh, November 2007	Storm surge	Up to 5.18 m	Agriculture	Cropland destroyed	0.65 million ha	Not specified
Odisha Super Typhoon, Odisha, India, October 1999	Storm surge	Up to 60 km inland from 480 km of shoreline	Agriculture	Farmland rendered infertile	200,000 ha	Not specified
Unnamed event, Solent, UK, March 2008	Storm surge	0.7 m of skew surge, flooding 7 km^2^ with up to 2.48 m of water	Buildings	Flooded and damaged	150 buildings, including at least 30 houses, 100 caravans, and a ferry terminal	Not specified
Hurricane Katrina, New Orleans, USA, August 2005	Storm surge	7.3 to 8.5 m high	Buildings	Inundated	80% of the city under 6m of water	21 days
Hurricane Katrina, New Orleans, USA, August 2005	Storm surge	Not specified	Buildings	Inundated	80% of the city, including 228,000 housing units	Not specified
Unnamed event, Avarua, Cook Islands, December 1967	Storm surge	Not specified	Buildings	Houses inundated	Affecting 270 residents	Not specified
Typhoon Haiyan, Tacloban City, Philippines, November 2013	Storm surge	Not specified	Buildings	Destroyed	All wooden constructions on the coastline	Not specified
Cyclone Meena, Avarua, Cook Islands, February 2005	Storm surge	Waves up to 14 m, surge reaching 360 m inland at 2 m above high tide mark	Buildings	Largely destroyed	Avarua Wharf	Not specified
Cyclone Sally, Avarua, Cook Islands, January 1987	Storm surge	Waves 10 m higher than normal	Buildings	Heavily damaged	Avatiu Harbor	Not specified
Cyclone Sally, Avarua, Cook Islands, January 1987	Storm surge	Waves 10 m higher than normal	Buildings	Damaged	Entire North Coast of Avarua	Not specified
Unnamed event, Avarua, Cook Islands, December 1831	Storm surge	Not specified	Buildings	Destroyed	Half the town	Not specified
Unnamed event, Avarua, Cook Islands, February 1935	Storm surge	200 m incursion, to >30 m beyond high tide mark	Buildings	Inundated	Lowland settlement	Not specified
Unnamed event, Avarua, Cook Islands, February 1935	Storm surge	200 m incursion, to >30 m beyond high tide mark	Buildings	Hospital and other buildings damaged	Lowland settlement	Not specified
Cyclone Meena, Avarua, Cook Islands, February 2005	Storm surge	Waves up to 14 m, surge reaching 360 m inland at 2 m above high tide mark	Buildings	Damaged	Much of North and Northwest coast	Not specified
Unnamed event, Ngatangiia, Cook Islands, January 1946	Storm surge	Not specified	Buildings	Church wall destroyed	1 church	Not specified
Cyclone Sally, Avarua, Cook Islands, January 1987	Storm surge	Waves 10 m higher than normal	Buildings	Shops inundated	1 commercial center	Not specified
Cyclone Sally, Avarua, Cook Islands, January 1987	Storm surge	Waves 10 m higher than normal	Buildings	Buildings damaged	One commercial center	Not specified
Unnamed event, Avarua, Cook Islands, December 1967	Storm surge	Not specified	Buildings	Damaged, buried	1 hotel	Not specified
Cyclone Sally, Avarua, Cook Islands, January 1987	Storm surge	Waves 10 m higher than normal	Buildings	Restaurant destroyed	1 restaurant	Not specified
Cyclone Heta, Avarua, Cook Islands, January 2004	Storm surge	10 m waves	Buildings	Inundated	Several areas	Not specified
Cyclone Meena, Avarua, Cook Islands, February 2005	Storm surge	Waves up to 14 m, surge reaching 360 m inland at 2 m above high tide mark	Buildings	Damaged	Several buildings	Not specified
Cyclone Nancy, Matavera, Cook Islands, February 2005	Storm surge	Not specified	Buildings	Inundated	Several buildings	Not specified
Cyclone Nancy, Ngatangiia Harbour, Cook Islands, February 2005	Storm surge	Not specified	Buildings	Damaged	Several buildings	Not specified
Unnamed event, Mid-Atlantic Coast, USA, 1962	Storm surge	Not specified	Buildings	Destroyed urban structures	Up to 32 km inland	Not specified
Unnamed event, Solent, UK, March 2008	Storm surge	0.7 m of skew surge, flooding 7 km^2^ with up to 2.48 m of water	Roads	Flooded	22 roads	Not specified
Cyclone Meena, Avarua, Cook Islands, February 2005	Storm surge	Waves up to 14 m, surge reaching 360 m inland at 2 m above high tide mark	Roads	Damaged	500 m of coast road	Not specified
Cyclone Sally, Avarua, Cook Islands, January 1987	Storm surge	Waves 10 m higher than normal	Roads	Destroyed	6 km of coastal road	Not specified
Cyclone Sidr, Sarankhola Upazi, Bangladesh, November 2007	Storm surge	1.5 m	Roads	Roads and embankments destroyed or damaged	85% of regional infrastructure	Not specified
Unnamed event, Avarua, Cook Islands, December 1967	Storm surge	Not specified	Roads	Eroded, buried	1 coastal road	Not specified
Cyclone Heta, Avarua, Cook Islands, January 2004	Storm surge	10 m waves	Roads	Inundated and damaged	1 seawall road	Not specified
Superstorm Sandy, New York, October 2012	Storm surge	4.3 m	Water	Damaged wastewater infrastructure	560 million gallons of untreated sewerage released	Not specified
